# Noninvasive assessment of fetal metabolic acidemia by automated analysis of cardiac function using color tissue Doppler imaging in a sheep model

**DOI:** 10.1038/s41598-026-66360-0

**Published:** 2026-08-10

**Authors:** Huan Liang, Jonas Johnson, Juulia Lantto, Heikki Huhta, Mervi Haapsamo, Merja Kokki, Hanna-Marja Voipio, Magnus Westgren, Amar Bhide, Juha Räsänen, Ganesh Acharya

**Affiliations:** 1 Division of Obstetrics and Gynecology, Department of Clinical Science, Intervention and Technology (CLINTEC), Karolinska Institutet, 14186 Stockholm, Sweden; 2 Shanghai Key Laboratory of Reproduction and Development and Shanghai Key Laboratory of Reproductive Endocrine Related Diseases, Obstetrics and Gynecology Hospital of Fudan University, Shanghai, 200433 China; 3 Center for Fetal Medicine, Karolinska University Hospital, Stockholm, Sweden; 4 Translational Research Group, Oulu University Hospital and Oulu University, Oulu, Finland; 5 Central Hospital of Central Ostrobothnia and Oulu University, Oulu, Finland; 6 Anesthesiology and Intensive Care, Kuopio University Hospital, Kuopio, Finland; 7 Faculty of Health Sciences, School of Medicine, University of Eastern Finland, Kuopio, Finland; 8 Oulu Laboratory Animal Centre, Oulu University Hospital and Oulu University, Oulu, Finland; 9 Fetal Medicine Unit, St George’s University Hospitals NHS Foundation Trust, London, UK; 10 Department of Obstetrics and Gynecology, Helsinki University Hospital and University of Helsinki, Helsinki, Finland; 11 Women’s Health and Perinatology Research Group, UiT – The Arctic University of Norway, Tromsö, Norway

**Keywords:** Cardiology, Diseases, Health care, Medical research, Physiology

## Abstract

Reliable noninvasive methods for accurately diagnosing fetal metabolic acidemia are still lacking. Our aim was to evaluate an ultrasound-based noninvasive method for assessing metabolic acidemia by an automated analysis of cardiac motion using color tissue Doppler imaging (cTDI) of the fetal heart in an experimental sheep model. Ten pregnant sheep and their fetuses were instrumented at 119–120 days gestation under general anesthesia. After a four-day recovery period, fetal arterial blood pressure, blood gases and heart rate were measured invasively. Cine-loops of cTDI images of the fetal heart were recorded at baseline and every 15 min for 3 h after uterine artery occlusion followed by epidural anesthesia and maternal hypo-oxygenation aiming to achieve a fetal arterial pH ≤  7.2 or base excess ≤ − 12 mmol/L or lactate > 4.8 mmol/L. The cTDI images were analyzed using an automated algorithm to measure myocardial velocities, cardiac cycle time intervals and atrioventricular plane displacement (AVPD). A Bayesian multivariate multilevel model was fitted to quantify associations between fetal cardiac function parameters and blood gas values. Data from eight fetuses were available for analyses. Among 21 predictors, left AVPD showed credible associations with fetal arterial pH, base excess and lactate and was the strongest and most robust single predictor with the association driven by within-fetus change over time rather than differences between animals. Left ventricular systolic peak velocity and right ventricular pre-ejection period showed similar but weaker associations, consistent with a shared myocardial mechanism rather than independent predictors. No other parameter showed credible associations with all three outcomes. Decreased left AVPD measured by automated analysis of cTDI cine-loops of the heart was associated with fetal acid–base status and may represent a candidate noninvasive marker of metabolic acidemia, warranting further validation.

**Supplementary Information:**

The online version contains supplementary material available at 10.1038/s41598-026-66360-0.

## Introduction

Hypoxemia is a common challenge fetuses face during intrauterine life, especially during labor and delivery. A substantial number of fetuses die or acquire permanent neurological damage due to hypoxic–ischemic encephalopathy (HIE) as a consequence of intrauterine hypoxia or birth asphyxia^1,2^, and its prevalence remains unchanged^3^. Although the outcome of intrapartum fetal hypoxemia is quite variable, there is an association between severity of fetal metabolic acidemia and HIE. The highest recorded lactate value in the first hour of extrauterine life is also shown to be an important predictor of moderate to severe HIE^4^.

The response of the cardiovascular system and fetal myocardium plays a central role in the adaptive mechanisms associated with hypoxia^5–9^. The progression of myocardial hypoxia/ischemia is initiated by impaired perfusion, later followed by metabolic changes leading to cardiac dysfunction^5^. Consequently, accurate assessment of fetal cardiac function during fetal cardiovascular adaptation to hypoxia could be useful for detecting and determining the degree of fetal hypoxia. Electronic fetal heart rate monitoring, that is currently used in clinical practice, has a very low predictive value in predicting metabolic acidemia^10^ or cerebral palsy^11–13^. Fetal umbilical cord sampling antenatally or intermittent fetal scalp blood sampling during labor to measure blood gases and acid–base status are the methods available today to diagnose fetal hypoxemia and acidemia in clinical practice. However, both are invasive, and a reliable noninvasive method for detecting fetal metabolic acidemia is still lacking.

Fetal cardiovascular function can be evaluated noninvasively using Doppler echocardiography^14–18^, but commonly used parameters of cardiac function are unchanged during fetal hypoxemia. In severely growth-restricted human fetuses with impaired placental circulation and no evidence of metabolic acidemia, the weight-indexed combined ventricular cardiac output is maintained at normal level^15,19^. In experimental settings, the sheep fetuses have been shown to maintain their ventricular cardiac outputs even during prolonged hypoxemia leading to metabolic acidemia, but changes in myocardial velocities and cardiac cycle time intervals could be detected using tissue Doppler imaging (TDI) in experimental settings^7,20^. However, manually measuring such parameters is operator dependent, time consuming and its clinical application is hampered by observer dependency and the cumbersome analysis process required to obtain reliable measurements. Recently, we have validated the use of an automated expert system for analyzing cine-loop images of the 4-chamber view of the fetal heart acquired using spectral color tissue Doppler imaging (cTDI) as a faster and reliable method of measuring myocardial velocities, cardiac cycle time intervals^21^ and atrioventricular plane displacement (AVPD)^22^ in human fetuses. In this experimental study we wanted to explore whether this noninvasive technique could detect changes associated with fetal metabolic acidemia.

We hypothesized that developing fetal metabolic acidemia would be associated with measurable changes in myocardial velocities, cardiac cycles time intervals and AVPD. In this experimental study we aimed to evaluate whether automated cTDI derived cardiac motion parameters are associated with hypoxemia/acidemia.

## Materials and methods

A total of 10 Åland Finish landrace time-dated pregnant sheep sourced from an accredited farm were purchased for the study through a commercial supplier (Lammastila SikkaTalu, 21,140 Rymättylä, Finland). The gestational age was 119–120 days (term 145 days) on the day of surgery. All experiments were performed in accordance with the European Union Directive (2010/63/EU) on protection of animals used for scientific purpose, corresponding Finnish regulations (Act 497/2013 on the protection of animals used in scientific and educational purposes), and the ARRIVE guidelines^23^. The research protocol was approved and the ethical permission to conduct the study was given by the National Animal Experiment Board of Finland (reference no. ESAVI/2387/04.10.07/2017).

### Preoperative care

After transporting to the animal laboratory center, the ewes were housed and cared according to the EU Directive, European Commission Recommendation of 18 June 2007 on Guidelines for the Accommodation and Care of Animals Used for Experimental and Other Scientific Purposes (2007/526/EC) and national legislation. After at least 7 days adaptation period, the ewe was fasted overnight before instrumentation with free access to water. On the day before surgery, the antebrachium of the ewe was shaved and cleansed with 70% ethanol. Transdermal fentanyl matrix patch (Fentanyl Ratiopharm®, Ulm, Germany) with a dose of 1.5 µg/h/kg was applied onto the dry skin and fixed in place with an adhesive tape. An elastic bandage was wrapped firmly around the antebrachium to give constant pressure on the patch. The patch was changed, and a new patch was applied after 72 h. On the day of surgery, the ewes received premedication with intramuscular ketamine (2 mg/kg) and midazolam (0.2 mg/kg) 30 min before the start of instrumentation for induction of anesthesia. The ewe´s external jugular vein was cannulated for intravenous access to administer of fluid and drugs. The auricular artery was cannulated to invasively monitor ewe´s heart rate and arterial blood pressure.

### Anesthesia

A standardized general anesthesia was used. Anesthesia was induced with a single bolus of intravenous propofol (4–7 mg/kg). Orotracheal intubation was performed with a cuffed tube. Anesthesia was maintained with sevoflurane (1–2.5%) in 40% oxygen-air mixture delivered via the endotracheal tube with positive pressure ventilation in a semi-closed circle system and propofol-infusion (3–5 mg/kg/h). Mechanical ventilation was provided, with the tidal volume adjusted to 10 ml/kg and the respiratory rate to 18/minute. After the induction of anesthesia, lumbar epidural catheter was inserted, and ewe was given epidural oxycodone bolus (0.1 mg/kg) followed by continuous infusion (0.1 mg/kg/h) during surgery. The ewes’ invasive arterial blood pressure, heart rate, peripheral oxygen saturation, anesthetic gas, oxygen and end-tidal CO2 partial pressure were monitored continuously (Carescape B650, GE Healthcare Finland, Helsinki, Finland) while under anesthesia. For intraoperative fluid management, the ewe was given an intravenous infusion of ringer-acetate 100 ml/h, and the flow rate was monitored with an electronic device (Monidror®, Monidor, Oulu, Finland).

### Surgery and instrumentation

**Fig. 1:**
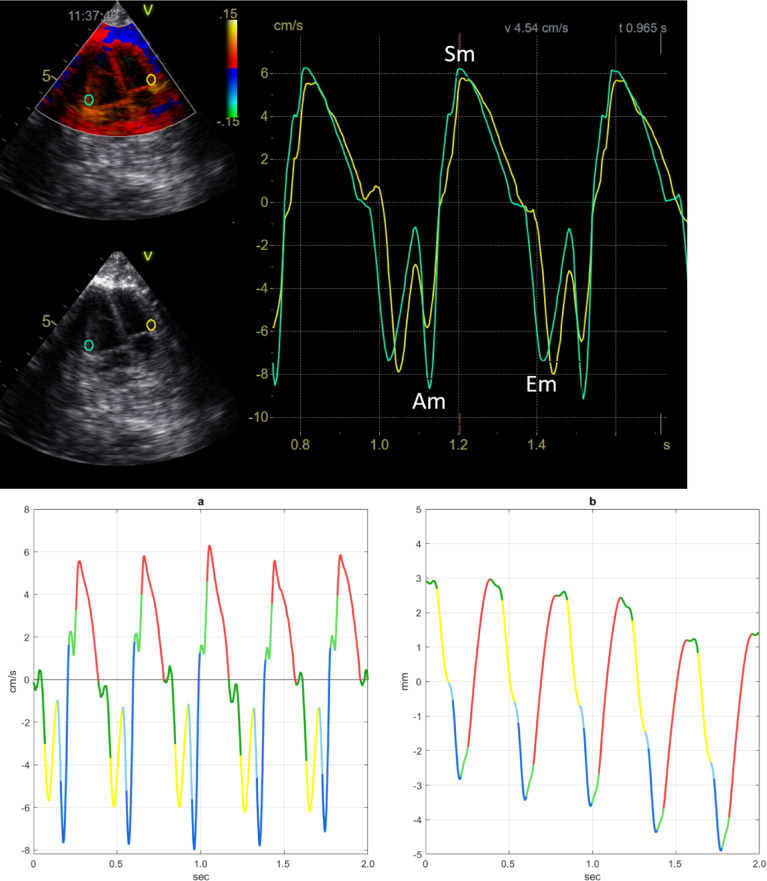
Measurement of fetal myocardial velocities and atrioventricular displacement using color tissue Doppler imaging (cTDI) of sheep fetal heart. Upper panel: Regions of interest were marked manually with (green circle = left ventricular wall and yellow circle = right ventricular wall) on a six-second cine-loop image clip of four-chamber view of the fetal heart using GE EchoPac software version 204. *Abbreviations: Sm, Systolic peak velocity, Em, Early-filling peak velocity, Am, Atrial-contraction velocity.* Lower panel: Automated analysis of atrioventricular plane movement from the left ventricular wall. (**a**) The myocardial velocity trace (cm/s), where an automated algorithm segments the waveform into distinct cardiac phases—atrial contraction (dark blue), pre-ejection (light green), ventricular ejection (red), post-ejection (dark green), and rapid (yellow) and slow (light blue) ventricular filling and determines the peak velocity within each phase. (**b**) The corresponding atrioventricular plane displacement (mm) over the same time duration.

The ewe was placed supine with a 30-degree lateral tilt. A midline laparotomy was performed in the lower abdomen to expose the uterus. An ultrasonic transit-time flow probe (Transonic Systems Inc., Ithaca, NY, USA) was placed around the uterine artery supplying the pregnant horn of the uterus and an inflatable cuff occluder (In Vivo Metric, Healdsburg, CA, USA) was placed slightly proximal to the probe and fixed in the positions using silk sutures (Fig. 1).

Thereafter, one of the fetal hind limbs was delivered through a small uterotomy incision. Femoral artery and vein were dissected, and polyvinyl catheters were inserted via each vessel to the descending aorta and inferior vena cava, respectively to obtain access to the fetal arterial and venous blood and measure blood pressures.

A separate polyvinyl catheter was placed in the amniotic cavity. The lost amniotic fluid was replaced with warm sterile saline solution. The fetus received penicillin G 1 million units either intravenously or via intra-amniotic catheter. Uterotomy and the abdominal incision were be closed, catheters and probes were exteriorized via a subcutaneous tunnel and fixed on sheep´s flank and flushed with heparinized saline. The skin at the incision site was infiltrated with 20 ml of bupivacaine (5 mg/ml) after closing the surgical wound.

### Postoperative care

The ewe was recovered from anesthesia after surgery and transferred to an individual pan. Post-operatively, the ewes had unlimited access to food and water after recovery from anesthesia. Transdermal fentanyl and epidural infusion of oxycodone (0.025 mg/kg/h) or oxycodone (0.015 mg/kg/h) with bupivacaine (1 mg/h) was used for postoperative pain management.

### Experimental protocol

After a 4-day recovery period experiments were be performed under general endotracheal anesthesia as described above, but without premedication. The depth of anesthesia was monitored and adjusted as required. Maternal femoral arterial pressure was measured continuously. Fetal arterial pressure was measured continuously via the femoral artery catheter (Biopac Systems Inc., Santa Barbara, CA, USA) inserted. Maternal and fetal mean arterial pressures (MAPs) were computed arithmetically (fetal arterial blood pressures were corrected by subtracting amniotic fluid pressure), and the heart rates (HRs) were computed from the arterial waveforms. Uterine artery blood flow (QUtA) was measured with the ultrasonic flow probe attached to a flow meter (T206, Transonic Systems Inc., Ithaca, NY, USA). All these variables were recorded continuously at a sampling rate of 1000-Hz using a polygraph (UIM100A, Biopac Systems Inc., Santa Barbara, CA, USA) and computerized data acquisition software (Acqknowledge v. 3.5.7 for Windows, Biopac Systems Inc., Santa Barbara, CA, USA).

At time zero when all hemodynamic parameters were stabilized, baseline measurement of fetal blood gases, were performed using an Abbot i‐Stat 1 blood gas analyser (i‐Stat, East Windsor, NJ, USA). Fetal hypoxia was induced by inflating the cuff of the uterine artery occluder. At 30 min, the ewe was given 5 ml of bupivacaine 0.5% through the epidural catheter as a test dose. After 2 min, bupivacaine 0.5% to a total dose of 0.3 ml/kg was administered slowly in 2 min. Hypotension to at least a 30% decrease in maternal systolic arterial pressure was allowed to develop. The hypoxia was gradually increased aiming to achieve fetal metabolic acidemia defined as arterial pH of ≤7.20 or BE of ≤ -12 mmol/L or lactate > 4.8 mmol/L, a level that is an indication for urgent delivery of the fetus in clinical practice. When the desired level of fetal metabolic acidemia was not reached after 60 or 120 min of starting uterine artery occlusions, maternal hypo-oxygenation was introduced by replacing maternal inhaled oxygen with medical air until maternal oxyhemoglobin saturation reached 80–90%.

Fetal arterial blood samples were obtained approximately every 15 min for 3 h or until fetus died due to severe acidemia, whichever came first. At the end of the experiment, the ewe and the fetus were euthanized by intravenous administration of pentobarbital sodium (Mebunat vet; Orion Oy) 100 mg/kg body weight to the ewe. The fetal weight was determined post-mortem.

### Echocardiography

Fetal echocardiography was performed by a single operator (JR) transabdominally using a GE Vivid-7 ultrasound system (GE; Vingmed, Horten, Norway) using a 10S transducer. Measurements were obtained at baseline and repeated every 30 min during hypoxemic challenge. Six second cine-loops of standard four-chamber view images of the heart in color tissue Doppler velocity imaging (cTDI) mode were recorded and stored for offline analysis. Image acquisition was optimized to obtain a high frame rate (> 150 frames/s).

cTDI images of the 4-chamber view of the fetal heart were analyzed using a dedicated software (EchoPac v204, GE Vingmed Ultrasound AS, Horten, Norway). Regions of interest (size 3 × 6 mm) were placed at the level of atrio-ventricular plane in the right and left ventricular walls. The data were then exported to MATLAB 2023b (MathWorks, MA, USA). An automated rule-based system combined with pattern recognition^21,22^ was used to analyze myocardial tissue motion at baseline and during different phases of experiment.

The cardiac cycle time intervals (atrial contraction, pre-ejection period, ventricular ejection, post-ejection period, early ventricular filling and mechanical PR interval i.e. atrio-ventricular conduction time), myocardial peak velocities at atrial contraction, ventricular systole, and early ventricular filling were measured automatically^21^. Myocardial performance index was calculated as: (pre-ejection period + post-ejection period)/ventricular ejection time. Atrioventricular plane displacement (AVPD) was also automatically measured as the maximum longitudinal distance of the atrioventricular plane movement from cardiac base to apex by the integration of myocardial systolic velocity^22,24^. Figure 1 demonstrates the placement of regions of interest on a cTDI image of the 4-chamber view of the fetal heart along with the traces representing myocardial velocities and displacement during the cardiac cycles. The myocardial motion parameters (velocity, displacement and time intervals) used for statistical analyses were measured from all available cardiac cycles recorded on a cine-loop image over a period of 6 s and then averaged.

## Data analyses

### Design and predictors

We employed a longitudinal mixed-effects modeling framework to investigate the relationship between repeated measures of fetal acid–base status (i.e., lactate, pH, and base excess) and several ultrasound-derived cardiac motion parameters. These features were measured in a cohort of eight lamb fetuses over 13 experimental time points.

To address missing data, we applied a systematic rule-based approach. First, predictors with excessive missingness (e.g., completely missing for more than two subjects or having < 60% overall completeness) were excluded. No data were missing for the primary predictor, left AVPD (AVPD-L). The remaining missing values were then imputed on a per-subject basis using Multivariate Imputation by Chained Equations (MICE). After this process yielded a complete dataset, two final preprocessing steps were performed. First, all continuous predictors (including Time and Heart Rate) were standardized (z-scored). Second, to account for temporal autocorrelation, one-step lagged, versions of each predictor were generated and standardized; a squared (quadratic) term was additionally generated for the quadratic candidate models.

All analyses were conducted using Python-based tools. Statistical modeling was performed using PyMC (version 5.21.1), with posterior diagnostics and model evaluation carried out using ArviZ (version 0.21.0). Data preprocessing and manipulation were conducted using pandas (version 2.2.3).

### Model Specification

For each cardiac motion parameter, a Bayesian multivariate multilevel model was fitted to quantify its association with the three biochemical outcomes (i.e. lactate, pH and base excess). These outcomes were modeled jointly using a multivariate Student-t distribution to provide robustness to outliers. Each model included fixed effects for the concurrent (non-lagged) parameter, its one-step lagged version, time, and heart rate. For the quadratic candidate model, a squared term for the concurrent predictor was additionally included. To capture individual differences, subject-specific random intercepts were incorporated for each outcome. The covariance structures for both the random intercepts and the residuals were modeled using an LKJ prior (η = 2.0) on the correlation matrices.

### Model selection

To determine the most appropriate functional form for each predictor’s relationship with the outcomes, we fitted and compared two competing models: a linear form (containing only the concurrent and lagged terms) and a quadratic form (which also included the squared term). Both forms also included time and heart rate as fixed effects, and the equations below are shown in simplified form.

a linear form

$$Y \sim \beta_{0} + \beta_{1} *x + \beta_{lag} *x_{lag}$$

and a quadratic form

$$Y \sim \beta_{0} + \beta_{1} *x + \beta_{2} *x^{2} + \beta_{lag} *x_{lag}$$

Model comparison was performed using leave-one-out cross-validation (LOO-CV), with models ranked by the expected log pointwise predictive density (ELPD). To encourage parsimony, the more complex quadratic model was selected only if it provided a substantial improvement in fit, defined as an ELPD difference greater than twice the standard error of that difference (ΔELPD ≥ 2 × SE(ΔELPD)).

### Priors and Bayesian inference

Weakly informative priors were assigned to all parameters. Population-level intercepts were given Normal priors centered at the empirical mean of each outcome. Coefficients for the fixed effects of time, HR, the concurrent predictor were assigned Normal (0, 2.0) priors, the quadratic term a Normal (0, 1.0) priors, while the lagged predictor received a more conservative Normal (0, 0.5) prior. The degrees of freedom parameter (ν) for the Student-t likelihood was assigned a Gamma (2, 0.1) prior. Models were fitted using the No-U-Turn Sampler (NUTS). We ran 4 parallel chains for 3,500 iterations, with the first 2,000 discarded as a warm-up, resulting in 6,000 posterior samples for each parameter.

### Model evaluation and inference

Model convergence was assessed by ensuring the potential scale reduction factor (R^) was below 1.01 for all parameters. Model fit was evaluated both visually, using Posterior Predictive Checks (PPCs), and quantitatively, using Bayesian p-values for the mean and standard deviation of each outcome. The final evaluation of a predictor’s utility was based on the posterior summary (mean, 95% Highest Density Interval [HDI]) of the concurrent slope from its single best-fitting model (linear or quadratic). A predictor was considered to have a credible association if the 95% HDI for the concurrent slope excluded zero. To distinguish longitudinal change within fetuses from baseline differences between animals, each predictor was additionally decomposed into within-subject and between-subject components.

## Results

### Study cohort

Ten ewes and their fetuses were instrumented. Two fetuses were excluded (one postoperative death before the day of experimentation and one arterial catheter failure), leaving eight fetuses (four male, four female) for experimentation. Maternal mean weight was 60.5 ± 5.0 kg, fetal weight 2470 ± 408 g.

### Baseline physiology

On the day of experiment, maternal baseline HR was 122 ± 14.2 beats min⁻^1^ and MAP 95.1 ± 14.4 mmHg. Uterine-artery blood flow to the pregnant horn was 420 ± 145 mL min⁻^1^. Table 1 summarizes key fetal variables of acid–base status and corresponding fetal echocardiographic parameters at baseline and experiment end.

**Table 1:** Descriptive statistics of sheep fetal heart rate arterial blood pressure, acid base status and echocardiographic parameters. Data are presented as median and range (min–max). T1 = Baseline measurement; T13 = Last measurement.

Invasively measured parameters	T1: Median (range)	T13 Median (range)	T1: Median (range)	T13 Median (range)
Mean arterial pressure (mmHg)	38.00 [27.00–43.00]	48.00 [30.00–62.00]		
Heart rate (bpm)	156.30 [137.50–179.65]	219.33 [166.84–246.54]		
pH	7.33 [7.22–7.44]	7.15 [6.93–7.27]		
Base excess (mmol/L)	2.00 [− 4.00–4.00]	− 9.50 [− 20.00–− 4.00]		
Lactate (mmol/L)	1.54 [0.98–3.61]	12.00 [8.30–16.53]		
Echocardiographic parameters	Left ventricles		Right ventricles	
Atrioventricular plane displacement (mm)	5.40 [4.02–6.47]	3.51 [2.75–5.27]	5.97 [3.84–7.99]	4.03 [2.16–6.35]
Myocardial performance index	0.72 [0.37–1.47]	0.83 [0.31–2.12]	0.72 [0.31–2.05]	1.24 [0.20–3.21]
Cardiac cycle time intervals				
Atrial contraction period (ms)	56.13 [47.94–72.36]	44.36 [12.06–82.25]	55.95 [48.17–81.59]	46.86 [28.93–98.87]
Pre-ejection period (ms)	26.25 [9.88–41.01]	32.03 [8.15–51.11]	35.77 [21.15–69.29]	31.61 [15.77–51.45]
Ventricular ejection period (ms)	140.50 [115.19–170.76]	114.60 [61.69–143.72]	134.85 [85.58–195.71]	104.85 [71.03–184.41]
Post-ejection period (ms)	67.80 [43.88–98.08]	68.16 [33.28–107.15]	65.43 [28.74–125.68]	74.89 [24.86–100.03]
Rapid ventricular filling period (ms)	67.83 [39.27–89.29]	48.01 [26.99–108.17]	66.04 [35.63–85.22]	63.17 [43.80–69.62]
Atrioventricular conduction time (ms)	77.28 [59.91–98.91]	73.72 [28.10–113.00]	91.67 [60.85–132.18]	78.16 [28.93–135.85]
Myocardial velocities				
Atrial contraction peak velocity (cm/s)	− 6.28 [− 8.76–− 0.84]	− 3.53 [− 8.03–− 0.83]	− 5.85 [− 10.42–− 1.79]	− 5.27 [− 5.76–− 4.78]
Pre-ejection peak velocity (cm/s)	0.76 [0.07–3.28]	1.19 [0.35–1.94]	2.24 [0.67–7.43]	1.76 [0.23–19.30]
Ventricular systolic peak velocity (cm/s)	5.09 [2.81–7.45]	4.46 [1.94–6.32]	5.23 [2.63–6.08]	5.21 [3.42–7.63]
Post-ejection peak velocity (cm/s)	1.30 [0.12–1.94]	2.16 [0.36–7.21]	2.26 [0.31–5.68]	0.77 [0.42–6.66]
Early ventricular filling peak velocity (cm/s)	− 4.47 [− 7.47–− 3.46]	− 5.14 [− 8.42–− 1.47]	− 6.58 [− 7.78–− 3.62]	− 9.37 [− 11.66–− 7.08]

### Data preprocessing

First, predictors were assessed for data completeness. Three right-ventricular parameters were removed from the initial set owing to low completeness of data: atrial-contraction peak velocity 49% complete), rapid ventricular filling period (51% complete), and early-filling peak velocity (59.6% complete).

Bayesian models were then fitted for the remaining predictors, and each model’s stability was evaluated using the Pareto-k diagnostic. Two additional predictors were excluded at this stage due to unreliable leave-one-out estimates (Pareto-k > 0.7). This resulted in the final set of 21 predictors used for the final main statistical evaluation. For each parameter we also report the normalized width of the 95% highest density interval (NHDIW), defined as the interval width divided by the absolute posterior mean. Because this ratio increases without bound as the estimated effect approaches zero, large values indicate an effect close to zero rather than an imprecise estimate.

### Longitudinal trends

**Fig. 2:**
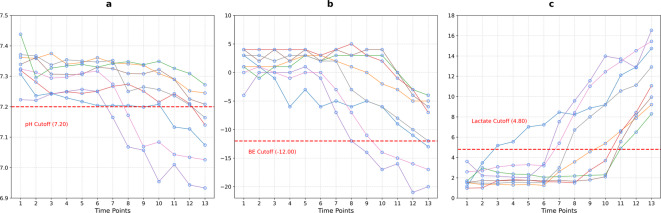
Temporal changes in biochemical markers of fetal acid–base status: pH (**a**), base excess (BE; **b**), and lactate (**c**) during the 13-steps of experiment. Each colored line represents the trajectory for an individual sheep fetus. The plots show a general decline in pH and BE and a corresponding rise in lactate after step 6, consistent with developing acidemia. Horizontal dashed red lines indicate the clinical cut-offs for pH (7.2), BE (-12 mmol/L), and lactate (4.8 mmol/L). The figure highlights the significant variation observed between subjects in both their baseline status and their rate of change.

**Fig. 3:**
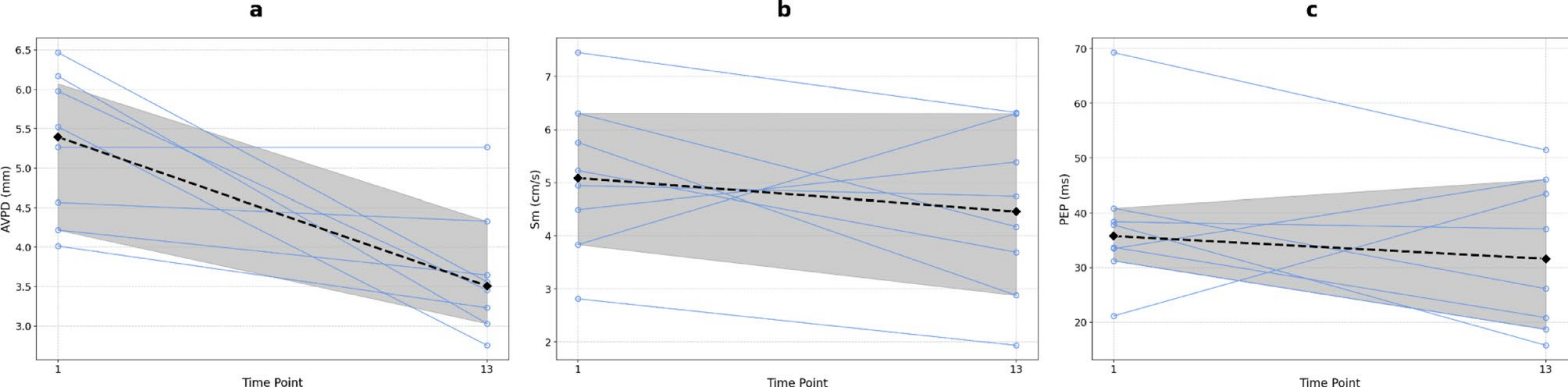
The plots display the changes in (**a**) atrioventricular displacement (AVPD) of the fetal left ventricular wall, (**b**) myocardial systolic peak velocity (Sm) of the fetal left ventricular wall and (**c**) pre-ejection period (PEP) of the right ventricular wall from baseline (left) to the last experimental time point (right). Each line represents the trajectory for an individual fetus (n = 8), and the bold, dashed black line shows the group median.

Figure 2 depicts individual trajectories of lactate, pH and BE, illustrating marked inter-subject variability under progressive hypoxemia and metabolic acidosis. Figure 3 shows the absolute change left AVPD (AVPD-L), left Sm and right ventricular wall pre-ejection period (PEP) from baseline to the last measurement before terminating the experiment.

### Predictor evaluation

Among the 21 predictors, the left AVPD (AVPD-L), Sm and the right ventricular PEP adjusted for heart rate emerged as robust markers of metabolic acidemia (Tables 2 and 3). The AVPD-L and the left ventricular wall Sm showed credible negative associations with lactate and positive associations with pH and base excess; the right-wall pre-ejection period (PEP) showed similar but weaker associations and was treated as secondary. In a within-subject versus between-subject decomposition, the AVPD-L association with lactate, pH, and base excess was credible in the within-subject component but not in the between-subject component, whereas the right AVPD (AVPD-R) showed no credible association (Supplementary Table S1).

**Table 2:** Posterior summary of the relationship between Left Ventricular cardiac parameters and fetal acid–base status. The table displays key statistics for the concurrent slope coefficient (β1) for each predictor. For each model, the posterior mean, its 95% Highest Density Interval (HDI), and its standard deviation (SD) are shown. Model fit and performance were assessed using Posterior Predictive Check *p*-values (PPC p) for the mean and standard deviation of the outcomes, and by Leave-One-Out Cross-Validation, reported as the Expected Log Pointwise Predictive Density (LOO-ELPD).

Predictor left wall	Outcome	Mean Slope (β₁)	95% HDI	SD (β₁)	NHDIW	PPC p (mean)	PPC p (std)	LOO_ELPD	LOO_p
Atrioventricular Plane Displacement (mm)	Lactate	− 0.130	[− 0.231 − 0.027]	0.05	1.57	0.59	0.86	− 24.93	41.53
	pH	0.020	[0.006 0.033]	0.01	1.35	0.56	0.34	− 24.93	41.53
	BE	1.617	[0.765 2.430]	0.42	1.03	0.57	0.36	− 24.93	41.53
Myocardial performance index	Lactate	− 0.010	[− 0.074 0.049]	0.03	12.30	0.57	0.82	− 33.00	40.75
	pH	0.009	[− 0.001 0.018]	0.01	2.11	0.54	0.38	− 33.00	40.75
	BE	0.267	[− 0.327 0.829]	0.30	4.33	0.54	0.40	− 33.00	40.75
Atrial contraction period (ms)	Lactate	0.061	[− 0.026 0.151]	0.05	2.90	0.56	0.82	− 35.01	42.09
	pH	− 0.011	[− 0.024 0.002]	0.01	2.36	0.57	0.36	− 35.01	42.09
	BE	− 0.411	[− 1.228 0.405]	0.41	3.97	0.59	0.39	− 35.01	42.09
Pre-ejection period (ms)	Lactate	0.020	[− 0.050 0.089]	0.04	6.95	0.56	0.82	− 35.91	41.39
	pH	− 0.005	[− 0.016 0.006]	0.01	4.40	0.54	0.37	− 35.91	41.39
	BE	− 0.476	[− 1.130 0.193]	0.34	2.78	0.55	0.40	− 35.91	41.39
Ventricular ejection period (ms)	Lactate	− 0.021	[− 0.128 0.084]	0.05	10.10	0.56	0.83	− 30.90	41.22
	pH	0.003	[− 0.012 0.018]	0.01	10.00	0.57	0.37	− 30.90	41.22
	BE	0.382	[− 0.504 1.298]	0.47	4.72	0.59	0.40	− 30.90	41.22
Post-ejection period (ms)	Lactate	− 0.059	[− 0.134 0.011]	0.04	2.46	0.56	0.81	− 33.49	42.15
	pH	0.007	[− 0.004 0.018]	0.01	3.14	0.55	0.37	− 33.49	42.15
	BE	0.539	[− 0.129 1.210]	0.34	2.48	0.57	0.39	− 33.49	42.15
Rapid ventricular filling period (ms)	Lactate	− 0.021	[− 0.090 0.049]	0.04	6.62	0.56	0.82	− 35.23	42.38
	pH	0.000	[− 0.010 0.011]	0.01	NA	0.56	0.37	− 35.23	42.38
	BE	0.233	[− 0.400 0.902]	0.33	5.59	0.57	0.40	− 35.23	42.38
Atrioventricular conduction time (ms)	Lactate	0.013	[− 0.078 0.103]	0.05	13.92	0.56	0.82	− 34.15	41.59
	pH	− 0.007	[− 0.020 0.007]	0.01	3.86	0.56	0.36	− 34.15	41.59
	BE	− 0.607	[− 1.420 0.235]	0.43	2.73	0.57	0.38	− 34.15	41.59
Atrial contraction peak velocity (cm/s)	Lactate	− 0.001	[− 0.071 0.078]	0.04	149.00	0.56	0.79	− 33.01	41.93
	pH	− 0.009	[− 0.020 0.003]	0.01	2.56	0.53	0.40	− 33.01	41.93
	BE	− 0.517	[− 1.205 0.170]	0.35	2.66	0.54	0.42	− 33.01	41.93
Pre-ejection peak velocity (cm/s)	Lactate	− 0.025	[− 0.089 0.042]	0.03	5.24	0.57	0.82	− 34.32	41.23
	pH	0.008	[− 0.001 0.018]	0.01	2.38	0.54	0.36	− 34.32	41.23
	BE	0.494	[− 0.094 1.048]	0.29	2.31	0.55	0.39	− 34.32	41.23
Ventricular systole peak velocity (cm/s)	Lactate	− 0.096	[− 0.170 − 0.021]	0.04	1.55	0.63	0.89	− 22.40	42.65
	pH	0.016	[0.006 0.027]	0.01	1.31	0.56	0.33	− 22.40	42.65
	BE	1.221	[0.568 1.923]	0.34	1.11	0.56	0.36	− 22.40	42.65
Early ventricular filling peak velocity (cm/s)	Lactate	0.092	[0.011 0.166]	0.04	1.68	0.54	0.79	− 27.83	42.10
	pH	− 0.009	[− 0.021 0.003]	0.01	2.67	0.58	0.35	− 27.83	42.10
	BE	− 0.350	[− 1.108 0.404]	0.39	4.32	0.58	0.38	− 27.83	42.10

BE: base excess, NHDIW: normalized highest density interval width.

**Table 3:** Posterior summary of the relationship between Right Ventricular cardiac parameters and fetal acid–base status. The table displays key statistics for the concurrent slope coefficient (β1) for each predictor. For each model, the posterior mean, its 95% Highest Density Interval (HDI), and its standard deviation (SD) are shown. Model fit and performance were assessed using Posterior Predictive Check p-values (PPC p) for the mean and standard deviation of the outcomes, and by Leave-One-Out Cross-Validation, reported as the Expected Log Pointwise Predictive Density (LOO-ELPD).

Predictor right ventricular wall	Outcome	Mean slope (β₁)	95% HDI	SD (β₁)	NHDIW	PPC p (mean)	PPC p (std)	LOO_ELPD	LOO_p
Atrioventricular plane displacement (mm)	Lactate	− 0.062	[− 0.166 0.039]	0.05	3.31	0.55	0.80	− 35.51	42.83
	pH	0.008	[− 0.008 0.022]	0.01	3.75	0.57	0.35	− 35.51	42.83
	BE	0.468	[− 0.465 1.398]	0.48	3.98	0.58	0.38	− 35.51	42.83
Myocardial performance index	Lactate	− 0.002	[− 0.077 0.067]	0.04	72.00	0.55	0.81	− 34.42	43.06
	pH	− 0.008	[− 0.018 0.004]	0.01	2.75	0.57	0.32	− 34.42	43.06
	BE	− 0.626	[− 1.260 0.067]	0.34	2.12	0.58	0.35	− 34.42	43.06
Atrial contraction period (ms)	Lactate	− 0.014	[− 0.120 0.081]	0.05	14.36	0.55	0.80	− 34.33	40.42
	pH	0.006	[− 0.008 0.021]	0.01	4.83	0.56	0.34	− 34.33	40.42
	BE	0.480	[− 0.434 1.364]	0.46	3.75	0.56	0.37	− 34.33	40.42
Ventricular ejection period (ms)	Lactate	− 0.006	[− 0.100 0.082]	0.05	30.33	0.55	0.80	− 34.30	41.22
	pH	0.003	[− 0.010 0.017]	0.01	9.00	0.56	0.36	− 34.30	41.22
	BE	0.113	[− 0.678 0.966]	0.42	14.55	0.57	0.39	− 34.30	41.22
Post-ejection period (ms)	Lactate	0.012	[− 0.071 0.099]	0.04	14.17	0.55	0.81	− 36.46	41.71
	pH	0.000	[− 0.013 0.012]	0.01	NA	0.56	0.37	− 36.46	41.71
	BE	− 0.138	[− 0.914 0.631]	0.40	11.20	0.56	0.39	− 36.46	41.71
Atrioventricular conduction time (ms)	Lactate	− 0.040	[− 0.110 0.034]	0.04	3.60	0.60	0.84	− 29.88	40.75
	pH	0.013	[0.002 0.023]	0.01	1.62	0.53	0.37	− 29.88	40.75
	BE	0.878	[0.233 1.534]	0.33	1.48	0.54	0.40	− 29.88	40.75
Post-ejection peak velocity (cm/s)	Lactate	0.025	[− 0.059, 0.109]	0.04	6.72	0.55	0.80	− 36.26	40.69
	pH	− 0.003	[− 0.016, 0.009]	0.01	8.33	0.55	0.37	− 36.26	40.69
	BE	− 0.185	[− 0.977, 0.582]	0.40	8.43	0.55	0.39	− 36.26	40.69
Ventricular systole peak velocity (cm/s)	Lactate	− 0.060	[− 0.143 0.021]	0.04	2.73	0.58	0.83	− 34.21	42.94
	pH	0.015	[0.003 0.027]	0.01	1.60	0.55	0.34	− 34.21	42.94
	BE	0.968	[0.223 1.761]	0.40	1.59	0.56	0.39	− 34.21	42.94
Pre-ejection period (ms)	Lactate	− 0.074	[− 0.136 − 0.011]	0.03	1.69	0.61	0.86	− 23.40	40.33
	pH	0.014	[0.005 0.023]	0.01	1.29	0.54	0.37	− 23.40	40.33
	BE	0.833	[0.267 1.424]	0.30	1.39	0.54	0.40	− 23.40	40.33

BE: base excess, NHDIW: normalized highest density interval width.

In accordance with our criteria, the one-step lagged term showed a credible association only for the pH outcome. Specifically, its 95% Highest Density Interval (HDI) excluded zero in the models for AVPD but not for other cardiac parameters.

**Fig. 4:**
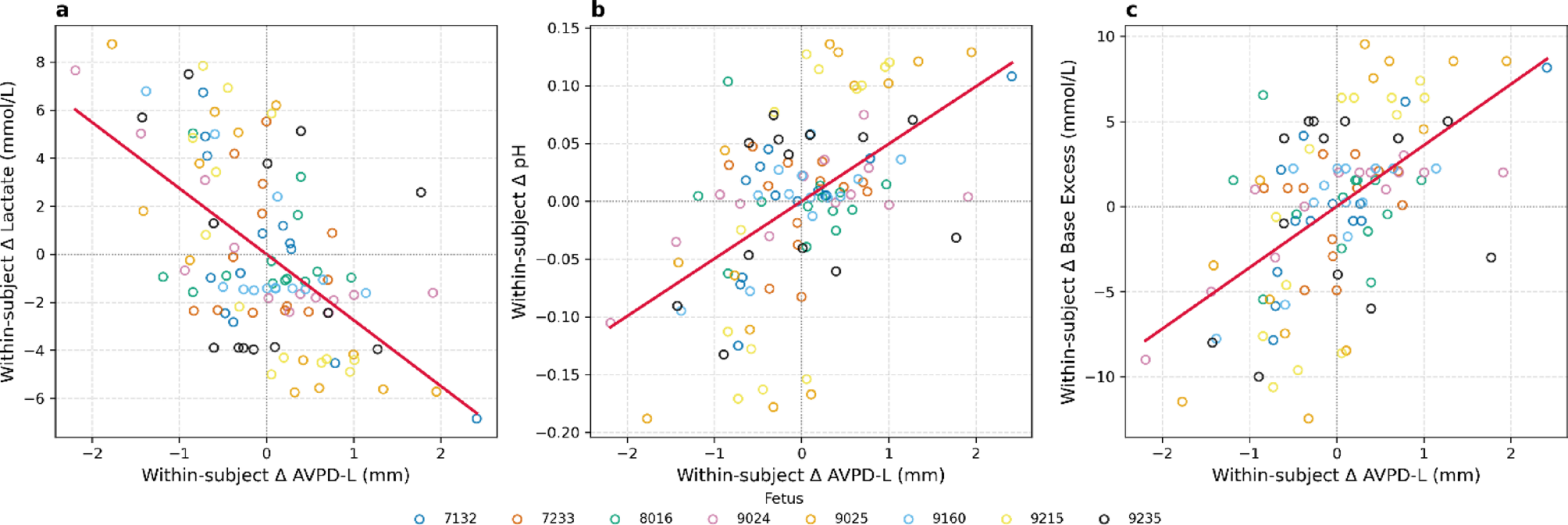
Within-subject relationship between left atrioventricular plane displacement (AVPD-L) and fetal acid–base status. For each fetus, AVPD-L and each outcome are expressed as deviations from that fetus’s own mean, thereby removing between-animal differences. The panels show the within-subject association between AVPD-L and (**a**) Lactate, (**b**) pH, and (**c**) Base Excess (BE). Each open circle represents one observation, and each fetus is represented by a different color (n = 8). The red line shows the fitted within-subject association. A decline in a fetus’s own AVPD-L is associated with higher lactate, and lower pH and base excess. The lactate axis is shown on the original, back-transformed scale (mmol/L).

**Fig. 5:**
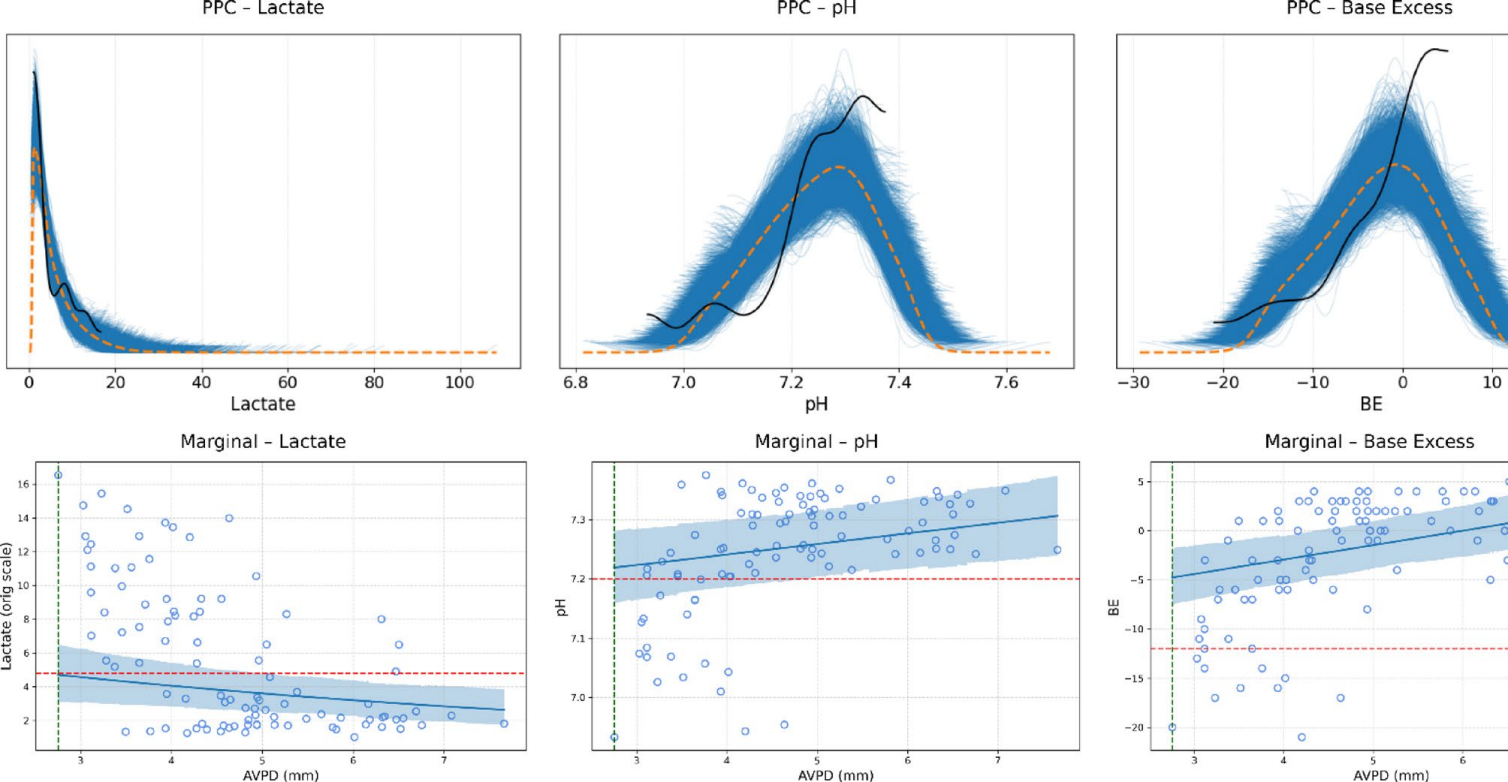
Posterior predictive checks and marginal effect plots for atrio-ventricular plane displacement (AVPD) of the left ventricular wall of the fetal heart. Top row: Posterior predictive distributions (blue lines) for Lactate, pH, and Base Excess (BE) under the linear model using AVPD as the predictor. The solid black line denotes the observed outcome distribution, while the orange dashed line shows the posterior predictive mean. The model captures central tendencies and spreads across all outcomes, although minor discrepancies are visible in the tails. Bottom row: Marginal effect plots of AVPD on each outcome (i.e. Lactate, pH and BE), adjusted for Time, Heart Rate, and subject-level intercepts. The solid blue line represents the posterior median prediction; the shaded region is the 95% Highest Density Interval (HDI). The green dashed line marks 2.8 mm, the lower end of the observed AVPD range, at which the predicted lactate approached the acidemia cutoff; it is shown in all three panels for reference. Horizontal red dashed lines indicate predefined acidemia reference thresholds used in the experimental protocol (Lactate > 4.8 mmol/L, pH < 7.20, and BE < -12 mmol/L).

Figure 4 demonstrates the relationship between AVPD-L and fetal acid–base status. AVPD-L was the strongest single predictor: β = − 0.130 for lactate (95% HDI − 0.23 to − 0.03), β = 0.020 for pH (95% HDI 0.006–0.033) and β = 1.617 for BE (95% HDI 0.765–2.430). Posterior predictive checks and marginal effect plots for AVPD-L are presented in Fig. 5. For lactate, the marginal prediction approached the acidemia cutoff (4.8 mmol/L) at the lowest AVPD-L values, corresponding to a critical value of approximately 2.8 mm (Fig. 5); the pH and base-excess predictions did not reach their respective cutoffs. Posterior mean slopes, uncertainty and LOO-ELPD were nearly identical for the three leading predictors, implying shared pathophysiology of impaired myocardial contractility rather than independent predictors.

## Discussion

This experimental study was performed to evaluate whether cTDI derived noninvasive fetal cardiac function parameters were associated with the level of fetal hypoxemia/acidemia determined by invasively measured biochemical markers (i.e. arterial pH, BE and lactate values). Our Bayesian multilevel analysis of 21 echocardiographic parameters identified three parameters most consistently associated with hypoxemia and acidemia. The decrease in the left AVPD, reduction of left ventricular wall Sm and shortening of the right-wall PEP adjusted for the fetal heart rate were associated with an increase in lactate and decrease in pH and base excess values (Tables 2 and 3). The PEP of the fetal heart has been shown to shorten during prolonged internal iliac artery occlusion and might be related to increased fetal blood pressure during hypoxemia caused by substantial reduction in uterine artery blood flow in pregnant sheep^25^. However, measures of left-sided fetal systolic cardiac function (AVPD-L and Sm) were the most informative, underscoring their value as the indicators of fetal compromise. It corroborates with the findings of our previous experimental study demonstrating that the fetal left ventricle is more sensitive to hypoxemia and metabolic acidemia compared to the right ventricle^26^.

Among the individual markers, decreased left AVPD was the single most powerful predictor, with credible associations with lactate (β = − 0.130, 95% HDI [− 0.23, − 0.03]), pH (β = 0.020, 95% HDI [0.006, 0.033]) and base excess (β = 1.617, 95% HDI [0.765, 2.430]). Reduced left ventricular filling due to reduced pulmonary venous return following pulmonary vasoconstriction observed during fetal hypo-oxygenation^27^ could be a mechanism associated with reduced AVPD, as it is a load-dependent parameter. However, as the left ventricular Sm, which is less affected by cardiac loading conditions^28^ was also reduced. In sheep fetuses, the left ventricular Sm remained unchanged even during acute occlusion of ascending aorta suggesting that Sm is not affected by increased afterload (^40^). Therefore, our findings likely reflect a worsening metabolic state of myocardium. In our study of near-term sheep, a critical AVPD-L value of approximately 2.8 mm (Fig. 5) marked the lower end of the observed range, at which the model’s predicted lactate approached the acidemia cutoff. This value was derived from the multilevel model’s adjusted marginal prediction (controlling for experimental time, fetal heart rate and each fetus’s baseline). Therefore, this critical threshold was lower than the average of AVPD measurements at the last experimental time point, as expected. Establishing any decision threshold would require larger sample size, normalization for gestational age/fetal size and further validation in human fetuses as the findings from animal studies may not be directly transferable to clinical setting due to species differences. However, it could be possible to validate our findings in human fetuses using automated measurement of AVPD with cTDI just before fetal scalp blood sampling to confirm or refute fetal distress in labor, and/or within a few minutes before delivery by cesarean section followed by umbilical cord blood sampling at birth.

A key insight from our data analyses is the high redundancy among the top predictors, as posterior mean slopes, uncertainty and LOO-ELPD values were nearly identical. This convergence suggests they track a common pathophysiological process of impaired myocardial function rather than providing independent information. The AVPD has been shown to correlate with stroke volume^29^ and contribute significantly to ventricular pumping in adults^30^. As the AV annulus moves towards the relatively fixed apex as a result of longitudinal fiber shortening during myocardial contraction^31^, it is likely to reflect cardiac contractility. In our study the fetal cardiac output was maintained during metabolic acidemia despite a decrease in AVPD, most likely due to the increase in fetal heart rate. AVPD has been shown to be superior to Sm to assess left ventricular systolic function in children^32^. However, AVPD is heart size dependent^33^ and is affected by gestational age^34^, which are important factors to consider as a single cutoff may not be appropriate for all gestational ages.

### Strengths and limitations

Animal experiments were planned and conducted in compliance with the three-R (reduction, refinement and replacement) principles. A priori sample size calculation was not performed since a formal power estimation is generally not applicable to exploratory animal studies. We had eight successful experiments and were able to obtain both fetal blood gases as well as echocardiographic measurements longitudinally at several time points during the experiment from the same animals, which provided us with a dense longitudinal dataset to explore within subject associations^35^.

Different methods of inducing fetal hypoxemia are available. A commonly used method is intermittent umbilical cord occlusion, which replicates variable fetal heart rate decelerations observed in labor. However, this method causes abrupt reductions in venous return and substantial changes in fetal cardiac loading condition directly affecting cardiac performance. Therefore, we chose to use uterine artery occlusion to simulate the hemodynamic effect of uterine contractions in labor, followed by epidural anesthesia that is commonly used for pain relief in labor. However, due to substantial collateral circulation between uterine horns, which is common in pregnant sheep (^40^ ), the utero-placental perfusion was maintained despite complete occlusion of uterine artery (confirmed by lack of measurable blood flow using ultrasonic flow probe) supplying the pregnant horn of the uterus unilaterally. Therefore, we often had to use maternal hypo-oxygenation after 60–120 min of starting uterine artery occlusion to achieve desired level of fetal acidemia. Placement of the cuff occluder more proximally around the uterine artery or the hypogastric artery may result in a more significant decrease in uteroplacental blood flow reducing the need for maternal hypo-oxygenation^36,37^.

Different causes of hypoxia may affect fetal cardiac function differently. The effect will also be modulated by fetal maturity, ability to respond to stress and severity of hypoxia/acidemia. Our study was carried out at a narrow gestational age window in near term sheep fetuses and therefore we have not normalized AVPD for the cardiac dimensions. Although, the sheep fetal heart function is fully mature at this stage in gestation, the AVPD is significantly affected by the heart size (Wandt et al. 1967)^33^, and the validity of our findings for all gestational ages should not be assumed. Therefore, it might be important to normalize AVPD by fetal heart dimension when using it to predict fetal hypoxia in preterm or growth restricted fetuses^24^. Because a valid normalization formula cannot be derived from this small near-term cohort, future clinical work should evaluate indexed AVPD, for example normalized to fetal cardiac dimensions, or gestational-age-specific reference values, before any threshold-based use.

We chose pH, lactate and BE, the most used parameters of fetal acid–base status in clinical practice, to determine predictive value of noninvasive echocardiographic variables to detect fetal acidemia and used their cutoffs that are generally used to make decision to deliver the fetus urgently due to fetal distress and similar thresholds in umbilical cord arterial blood at births have shown to predict of adverse neonatal outcome^38,39,40,41^.

Fetal AVPD and cardiac cycle time intervals can be measured using different techniques, such as M-mode, pulsed-wave TDI, cTDI^24^. However, cTDI cine-loops of four chamber view of the fetal heart have some advantage as they are easier to record, have higher resolution, and several cardiac cycles can be analyzed quickly and measurements averaged using automated algorithm.

Occasionally, the automated algorithm had difficulty in reliably identifying the specific cardiac events and phases from the right ventricular wall trace resulting in missing data for a few parameters. The velocity traces from the right and left ventricles were often quite different. The right-sided trace could appear more sinusoidal (like a sine wave) and frequently lacked the sharp, clear transition points between different cardiac phases that were seen on the left side, making them harder for the algorithm to separate, especially when the fetal heart rate was high.

The primary predictor, AVPD-L, had no missing values and did not rely on imputation, although some other missing values in retained predictors were imputed using per-subject MICE. However, parameters with low completeness were excluded, which limits inference about right-sided phase parameters. The small number of animals precluded an independent hold-out validation set, and leave-one-out cross-validation should therefore be interpreted as an internal model check rather than external validation. The findings of our exploratory study are hypothesis-generating and require confirmation in adequately powered experimental studies and further clinical validation.

## Conclusion

Decreased left AVPD quantified through automated analysis of cTDI cine-loops of four-chamber view of the fetal heart was associated with fetal metabolic acidemia in near term sheep. Larger independent studies with normalization for fetal size and gestational age, and validation in human fetuses are required before clinical application.

## Supplementary Information

Below is the link to the electronic supplementary material.

Supplementary Material 1

## Data Availability

Data supporting the results reported in this paper can be made available to researchers by the corresponding author on receiving a reasonable request explaining the purpose.

## Code availability

The Python/Bayesian analysis code used for missing-data handling, multivariate Bayesian multilevel modelling, posterior predictive checks, model diagnostics, and generation of the reported statistical outputs has been deposited in Zenodo: 10.5281/zenodo.21443864
