# Supplementary material for: Noninvasive assessment of fetal metabolic acidemia by automated analysis of cardiac function using color tissue Doppler imaging in a sheep model
Source: Sci Rep. 2026 Aug 10;16:24739. doi: 10.1038/s41598-026-66360-0 (PMC13457862; doi:10.1038/s41598-026-66360-0)
Supplement: Supplementary file 1 [file 41598_2026_66360_MOESM1_ESM.pdf]

Supplementary Material

Noninvasive assessment of fetal metabolic acidemia by automated analysis of cardiac function using color tissue Doppler imaging in a sheep model

Supplementary Table S1. Within-subject versus between-subject decomposition of the AVPD associations

Each predictor was decomposed into a within-subject component (deviation from the subject mean) and a between-subject component (subject mean), both entered in the model (Mundlak approach), for left (AVPD-L) and right (AVPD-R) atrioventricular plane displacement. Values are posterior mean slopes with 95% highest density intervals (HDI). A within-subject association credibly different from zero indicates that a change in a fetus's own AVPD tracks a change in its acid-base status, independent of between-animal differences.

**Predictor**	**Outcome**	**Within-subject beta [95% HDI]**	**Between-subject beta [95% HDI]**	**Within excl. 0**
**AVPD-L**	Lactate	-0.113 [-0.214, -0.005]	-0.268 [-0.657, 0.133]	yes
**AVPD-L**	pH	0.019 [0.004, 0.033]	0.014 [-0.065, 0.091]	yes
**AVPD-L**	BE	1.55 [0.65, 2.36]	1.42 [-1.31, 3.74]	yes
**AVPD-R**	Lactate	-0.054 [-0.151, 0.056]	-0.136 [-0.570, 0.311]	no
**AVPD-R**	pH	0.008 [-0.007, 0.023]	-0.009 [-0.086, 0.071]	no
**AVPD-R**	BE	0.42 [-0.55, 1.35]	0.70 [-1.98, 3.41]	no

For AVPD-L, the within-subject component excluded zero for all three outcomes while the between-subject component did not, supporting a longitudinal within-fetus association. AVPD-R showed no credible association in either component. Multivariate Student-t multilevel models with correlated random intercepts, as in the main analysis; n = 96 observations from 8 subjects; within and between components orthogonal by construction; maximum R-hat = 1.000.

Supplementary Table S2. Per-parameter data completeness before imputation

Completeness of each evaluated cardiac parameter in the pre-imputation dataset (n = 104 observations). Parameters with less than 60% overall completeness, or completely missing in more than two subjects, were excluded; retained parameters with incomplete data were completed by per-subject chained-equation imputation. Among the 23 retained cardiac parameters, 26 of 2392 values (1.1%) were imputed. The primary marker (AVPD-L) and left systolic peak velocity (Sm) were fully observed, so the primary finding and the within-subject decomposition do not rely on imputation.

Parameter	Completeness	Status
**Left atrioventricular plane displacement (AVPD), primary marker**	100%	Retained (fully observed)
**Left systolic peak velocity (Sm)**	100%	Retained (fully observed)
**Right atrioventricular plane displacement (AVPD)**	100%	Retained (fully observed)
**Right pre-ejection period (PEP)**	97.1%	Retained
**All other retained parameters (n = 19)**	96.2-100%	Retained
**Right early-filling peak velocity (Em)**	59.6%	Excluded (<60% complete)
**Right rapid ventricular filling period**	51.0%	Excluded (<60% complete)
**Right atrial-contraction peak velocity (Am)**	49.0%	Excluded (<60% complete)
